# An 8-Week Ketogenic Diet Alternated Interleukin-6, Ketolytic and Lipolytic Gene Expression, and Enhanced Exercise Capacity in Mice

**DOI:** 10.3390/nu10111696

**Published:** 2018-11-07

**Authors:** Sihui Ma, Qingyi Huang, Takaki Tominaga, Chunhong Liu, Katsuhiko Suzuki

**Affiliations:** 1 Graduate School of Sport Sciences, Waseda University, Tokorozawa 359-1192, Japan; masihui@toki.waseda.jp (S.M.); hqyaaaaaa@163.com (Q.H.); 2 College of Food Science, South China Agricultural University, Guangzhou 510642, China; t.tominaga7713@gmail.com; 3 The Key Laboratory of Food Quality and Safety of Guangdong Province, Guangzhou 510642, China; 4 Faculty of Sport Sciences, Waseda University, Tokorozawa 359-1192, Japan

**Keywords:** ketogenic diet, keto-adaptation, lipid metabolism, IL-6

## Abstract

Adjusting dietary fat intake is reported to affect mitochondrial biogenesis and fatty acid oxidation (FAO), and thus may enhance exercise capacity. However, a high-fat diet where carbohydrate intake is not limited enough also makes it difficult for athletes to maintain weight, and may fail to force the body to utilize fat. As such, a low-carbohydrate, high-fat, ketogenic diet (KD) may be viable. We have previously reported that an eight-week KD enhances exercise capacity, and suggested the mechanism to be enhanced lipolysis and ketolysis. In the present study, we investigated how an eight-week KD alters mRNA expression during fatty acid mobilization, FAO and ketolysis. We found that an eight-week KD may remodel the lipid metabolism profile, thus contributing to influence exercise capacity. We also found that ketolysis, lipolysis and FAO adaptations may contribute to enhanced exhaustive exercise performance. Along with enhanced FAO capacity during exhaustive exercise, a KD may also alter IL-6 synthesis and secretion profile, thus contribute to fatty acid mobilization, ketolysis, lipolysis and preventing muscle damage. Both the lipid metabolism response and IL-6 secretion appeared to be muscle fiber specific. Taken together, the previous and present results reveal that an eight-week KD may enhance exercise performance by up-regulating ketolysis and FAO ability. Therefore, a KD may have the potential to prevent muscle damage by altering IL-6 secretion profile, indicating that a KD may be a promising dietary approach in endurance athletes, sports, and for injury prevention.

## 1. Introduction

Carbohydrates, lipids and proteins are three classes of molecules utilized as fuel, or to make up bodily structures. Among these classes, protein is a vital nutrient that holds our bodies together. Compared with protein, carbohydrates and lipids are more flexible for use as energy sources. Compared with the storage capacity for carbohydrates (which is limited to ~5 g glucose in blood circulation and ~100 g or ~500 g glycogen in skeletal muscle or liver), the upper limit for human lipid store as fat seems to be unrestricted [1]. A person weighing 70 kg, possessing 15% body fat percentage, is capable of completing >30 marathon races while utilizing stored fat [2]. Therefore, due to limited carbohydrate reserves but an abundant lipid reserve, coaches and elite athletes are wondering if there is an effective way to enhance fat utilization. Investigations into this started decades ago, and continue to the present day [3,4]. 

Excess adipose tissue may cause health problems, whilst excessive bodyweight can be a problem in some events that are divided by weight, e.g., Taekwondo [4]. Moreover, participants of extremely strenuous aerobic events, such as marathon runners, usually display very low body fat percentage (6.4 ± 1.3%, for males, according to a survey conducted in elite athletes in America) [5]. Therefore, for athletes, only pursuing an abundant lipid reservoir may be unrealistic, making the ability to utilize fat effectively more important. 

An attempt to increase dietary fat and enhance fat utilization proved ineffective in a human trial of young men provided with a fat supplement (987 ± 55 kcal/day) over 36 h, which did not alter 24 h energy expenditure (2783 ± 232 kcal/day vs. 2820 ± 284 kcal/day), and fat oxidation rate (fat oxidation 1032 ± 205 kcal/day vs. 1042 ± 205 kcal/day) [6]. However, a high-fat diet (HFD) combined with exercise enhanced fat oxidation, according to previous studies [7,8,9]. In studies conducted in both trained and untrained subjects, training increases fat oxidation in human subjects and reduces the reliance on carbohydrate as an energy source during submaximal exercise tests [7,8,9]. Researchers also report that HFD adaption, including keto-adaption, may enhance endurance capacity in trained cyclists and rodents [10,11]. To date, many studies have made it clear that an HFD is a two-sided coin. For example, a one-month HFD was reported to cause increased muscle mitochondrial biogenesis and insulin resistance (IR) at the same time [12]. In another study conducted in rodents, a one-month alternate-day HFD increased mitochondrial enzyme activities and protein content; however, a reduced oxidative capacity was also observed in a short period, e.g., genes of the electron transport chain, mitochondrial carrier proteins and mitochondrial biogenesis [13,14]. Clinical evidence shows that non-obese subjects are capable of adjusting fat oxidation in response to increased fat intake within a week, even when energy balance is sustained [15]. Therefore, long-term traditional HFD is challenging when employed as a fat-loading tactic, since it may cause a weight burden for athletes, along with health problems. That is why we employed a low-carbohydrate, high-fat, ketogenic diet in our previous study [10]. 

In our previous study, we reported that an eight-week low-carbohydrate, high-fat, ketogenic diet enhanced exercise capacity, but the mechanisms remained unclear. In the previous study, body fat content was not altered significantly by a ketogenic diet (KD), although the average weight of the KD mice dropped dramatically in the feeding period. Therefore, we postulated whether another form of fat reserve contributed to this enhancement. Intramuscular triacylglycerol (IMTG) is a special way for skeletal muscle to store lipids. During exercise, IMTG may constitute up to 20% of total energy turnover, thus contributing significantly to adenosine triphosphate (ATP) synthesis during exercise [16]. However, abnormal or excessive fat deposition in skeletal muscle may also induce insulin resistance [17]. Regulating intramuscular lipolysis is crucial for energy supply during exercise. Amati and colleagues reported that well-trained athletes exhibit higher levels of IMTG and diacylglycerol (DAG), together with a well-preserved sensitivity to insulin, indicating lipolysis may be enhanced during exercise [18]. Ketone bodies and fatty acids are produced or released from the liver and adipose tissue, where other organs may then employ the metabolites as an energy source during fasting, exercise or other medical conditions. It is reported that working muscles have an increased capacity to extract ketone bodies from circulating blood during exercise [19]. However, metabolic profiles of different muscle fiber types are reported to be different, which may be attributed to their function [20,21]. Therefore, it is reasonable to presume that enhanced fat utilization, (including fatty acid mobilization from adipose tissue and IMTG), transportation, and oxidation by different muscle fibers together with ketone body utilization can be directly linked to exercise capacity. In this present study, we studied the rate-limiting enzymes during FAO, to see whether an eight-week KD enhanced FAO and contributed to exercise capacity. 

Endurance exercise may induce systemic inflammatory response [22]. According to the above studies, plasma interleukin (IL)-6 may increase over 100-fold after strenuous exercise [23,24]. Muscle-derived, exercise-induced IL-6 is reported to have lipolytic properties [25]. However, whether IL-6 may contribute to keto-adaption, thus enhancing exercise capacity, is unknown. Since our previous report indicated the amount of fatty acid and triglyceride consumption were enhanced in KD mice, we have herein examined the association between IL-6 and enhanced lipolysis. As discussed previously, a KD has been investigated as a low-carbohydrate nutritional approach within athletic nutrition to performance enhancement, but controversial conclusions have been obtained [10,26,27]. In our previous studies, we reported that an eight-week, low-carbohydrate, ketogenic diet increased running time until exhaustion in male C57BL6/J mice, and suggest the mechanism to be an enhanced fat utilization [10,27]. In the present study, we have investigated any alteration in the pattern of messenger RNAs related to lipid mobilization, fatty acid utilization and ketone body oxidation, in red slow-twitch and white fast-twitch muscle tissues, and adipose tissue, to ascertain the underlying mechanisms.

## 2. Materials and Methods

### 2.1. Mouse Maintenance and Diets

Male C57BL/6J mice (*n* = 35) were purchased from Takasugi Experimental Animals Supply (Kasukabe, Japan) at 7 weeks of age, and were allowed to adapt to the environment for a week before formal experimentation commenced. Four or five animals were housed together to a cage (27 × 17 × 13 cm) in a controlled environment under a light–dark cycle (lights on at 08:00 and off at 20:00). The experimental procedures were approved and followed the Guiding Principles for the Care and Use of Animals in the Academic Research Ethical Review Committee of Waseda University (10K001). All mice were randomly divided into four groups: chow diet (control: Con), involving a chow diet and a promotion of sedentary behavior (*n* = 8), chow diet plus exercise (Con + Ex, *n* = 9. Ex is the abbreviation for exercise), a KD and a promotion of sedentary behavior, (*n* = 9), and a KD plus exercise (KD + Ex, *n* = 9). The KD diet (TP-201450) consisting of 76.1% fat, 8.9% protein and 3.5% carbohydrate, 7.342 kcal/g and the chow diet (AIN93G) consisting of 7% fat, 17.8% protein and 64.3% carbohydrate, 3.601 kcal/g) *wt*/*wt* were obtained from Trophic (TROPHIC Animal Feed High-tech Co., Ltd., Jiangsu, China). Mice were maintained on ad libitum chow diet or KD, for 8 weeks commencing at 8 weeks of age. 

### 2.2. Endurance Capacity Test Protocol

One week before exhaustive exercise, all mice were accustomed to the treadmill by running at 15 m/min for 10 min. The endurance test was performed on a motorized treadmill (Natsume, Kyoto, Japan). That is, mice in the Con + Ex and KD + Ex groups were subjected to treadmill running at 10 m/min for 15 min, followed by 15 minutes at 15 m/min and then 20 m/min, followed finally by running at 24 m/min and 7% grade until exhaustion. Exhaustion was defined as the inability to continue regular treadmill running despite the stimulation of repeated tapping on the back of the mouse. The running time of exercised mice was recorded. Immediately after the exhaustion, mice were sacrificed under light anesthesia with the inhalant isoflurane (Abbott, Tokyo, Japan). Heparinized blood samples were collected from the abdominal aorta under isoflurane-induced mild anesthesia, whilst tissues and organs were immediately excised and frozen in liquid nitrogen. Plasma was obtained from blood samples by centrifugation at 1500× *g* for 10 min at 4 °C. These samples were stored at −80 °C until analyses.

### 2.3. Real-Time PCR

Total RNA was extracted from the gastrocnemius muscle (white, fast-twitch muscle) and soleus muscle (red, slow-twitch muscle) using the RNeasy Fibrous Mini Kit, and from epididymal adipose tissue using the RNeasy Lipid Tissue Mini Kit (Qiagen, Valencia, CA, USA) according to the manufacturer’s instructions. The purity and concentration of total RNA was assessed using the NanoDrop system (NanoDrop Technologies, Wilmington, DE, USA). Total RNA was reverse transcribed to cDNA using the High Capacity cDNA Reverse Transcription Kit (Applied Biosystems, Foster City, CA, USA) according to the manufacturer’s instructions. PCR was performed with the Fast 7500 real-time PCR system (Applied Biosystems) using the Fast SYBR^®^ (Applied Biosystems) Green PCR Master Mix (Applied Biosystems). The thermal profiles consisted of 10 min at 95 °C for denaturation followed by 40 cycles of 95 °C for 3 s and annealing at 60 °C for 15 s. 18 s mRNA was used as the housekeeping gene, and the *ΔΔCT* method was used to quantify target gene expression. All data are represented relative to its expression as fold change based on the values of the Con group. 

### 2.4. ELISA Procedure and Glycerol Assay

Plasma and gastrocnemius IL-6 concentrations were measured using a R&D Mouse IL-6 ELISA Duo set (R&D Systems, Minneapolis, MI, USA) according to the manufacturer’s instructions. Gastrocnemius IL-6 concentration was related to total protein concentration measured using the Pierce™ BCA Protein Assay Kit (Thermo Fisher Scientific, Rockford, IL, USA) according to the manufacturer’s instructions. Plasma glycerol was measured using the Glycerol Colorimetric Assay Kit (Cayman Chemical Co., Ann Arbor, MI, USA).

### 2.5. Statistical Analysis

Data are presented as means ± standard deviation (SD). A two-way analysis of variance (ANOVA) was performed to determine the main effects of diet and/or exercise. Statistical analysis was done using Graphpad 7.0 (Graphpad, Ltd., La Jolla, CA, USA). When this analysis revealed significant interaction, Tukey’s post hoc test was performed to determine the significance among the means. Associations among variables were analyzed using Pearson’s correlation coefficient. Statistical significance was accepted as *p* < 0.05.

## 3. Results and Discussion

### 3.1. A Review of Endurance Capacity Test and Plasma Non-Esterified Fatty Acids and β-Hydroxybutyrate Concentration

Endurance capacity, non-esterified fatty acids (NEFA) and β-hydroxybutyrate (β-HB) concentration have been measured and reported previously [10]. In brief, normal-fed (chow) mice ran up to 243 ± 60 min until exhaustion, whereas KD-fed mice achieved a longer running time of 289 ± 67 min (Con + Ex vs. KD + Ex, *p* < 0.05), indicating an enhanced endurance capacity. Plasma NEFA increased from 1.3 ± 0.24 µEq/L to 2.4 ± 0.65 µEq/L in the chow-fed group indicating exercise-induced lipolysis, but the plasma NEFA might not have been effectively taken up and utilized by muscle cells [10]. On the contrary, plasma NEFA decreased from 2.2 ± 0.65 µEq/L to 1.5 ± 0.43 µEq/L in KD-fed mice, indicating that NEFA uptake and utilization was used effectively [10]. A significant difference was observed between the chow- and KD-fed mice’ NEFA concentrations. At baseline, β-HB was increased by nearly 10-fold in KD, from 0.29 ± 0.038 mmol/L to 2.4 ± 0.64 mmol/L [10]. However, this figure was 0.72 ± 0.10 mmol/L after exhaustive exercise, indicating ketolysis was enhanced by the 8-week KD. Therefore, the following complimentary analyses were undertaken in this investigation.

### 3.2. Plasma IL-6 Concentration and Exercise-Induced IL-6 mRNA Alternation in Both Slow- and Fast-Twitch Muscles 

**Figure 1:**
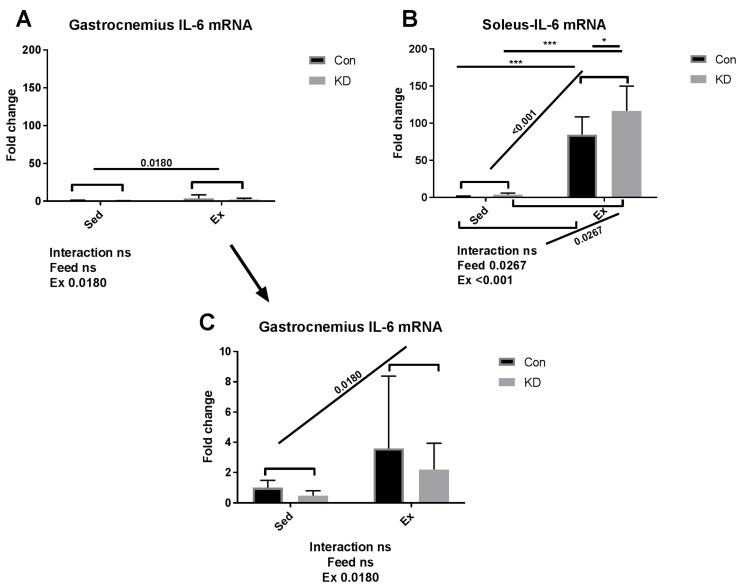
Interleukin-6 (IL-6) mRNA expression in gastrocnemius (**A**) and soleus (**B**) immediately after exhaustion. (**C**) is an amplified version of (**A**). ns: no significance observed. * *p* < 0.05 and *** *p* < 0.001. Significance between groups are marked using slashes.

As shown in Figure 1, IL-6 mRNA increased dramatically by approximately 100-fold in red, slow-twitch muscle fiber in the Con + Ex group; and in the KD + Ex group, a KD contributed to this interesting up-regulation. In the context of exhaustive exercise, transcription levels of IL-6 increased significantly in the KD + Ex group, compared to the Con + Ex group in red, slow-twitch muscle. However, the effect was not observed in white, fast-twitch muscle. The result indicated that IL-6 gene expression exhibited a fiber type specificity. Red, slow-twitch muscle fiber contributes more to endurance feats such as distance running; whereas white, fast-twitch muscles are used in powerful bursts of movements like sprinting, and they fatigue faster [20]. We found that the difference in fiber structure and function might lead to different secretion patterns of IL-6. An interesting heterogeneous phenomenon was observed during exercise; the subjects who exhausted around 200 min have the highest IL-6 gene expression (gastrocnemius muscle) and plasma IL-6 concentration in both groups. One reason may be that as exercise progresses, the need for fatty acids increases; however, as final fatigue is reached, the call for energy decreases with time. 

**Figure 2:**
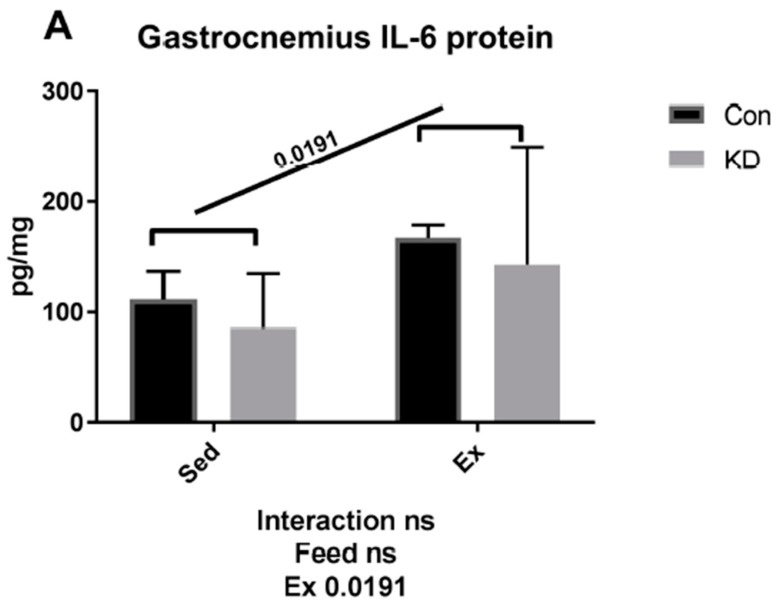

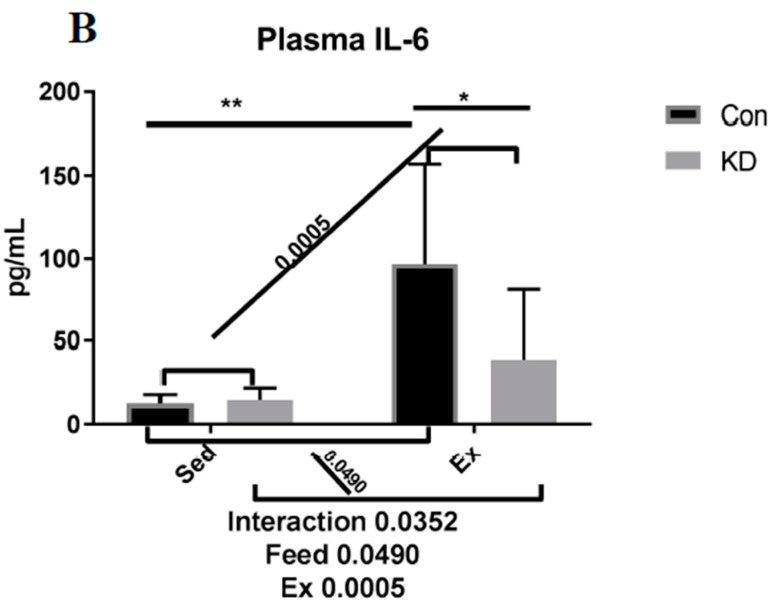
Interleukin-6 (IL-6) concentration in gastrocnemius (**A**) and plasma (**B**) immediately after exhaustion. ns: no significance observed. * *p* < 0.05 and ** *p* < 0.01. Significance between groups are marked using slashes.

As shown in Figure 2, both muscle IL-6 protein and plasma IL-6 were increased by exhaustive exercise. However, plasma IL-6 was significantly lower in the KD + Ex group, though IL-6 rose nearly 5-fold in the control feed group; it only increased to 2.5-fold in the KD group after exercise. A plausible explanation for this response is that the KD mice have built a well-adapted lipid-centered metabolism, and enhanced metabolic flexibility. It is worth mentioning that according to our previous study, circulating lipids, including ketone body, NEFA and triglyceride (TG), were all increased by a 2-month KD administration [10]. This abundant lipid reservoir may help weaken the need to accelerate lipolysis. 

Exercise-induced IL-6 is reported to stimulate lipolysis both in the IMTG pool and adipocytes [25,28,29]. Defined as an exercise factor by some researchers or a so-called myokine, muscle-derived IL-6 exhibits a metabolic regulation function towards lipid metabolism in different subject types [28,29]. Recombinant human IL-6 infusion showed enhanced lipolysis and fat oxidation capacity in human subjects [28]. Genetically IL-6 deficient mice also presented a reduced lipolysis ability and FAO [29]. During KD administration, fat oxidation is no doubt the predominant origin of energy supply which makes us suspect that acute exercise-induced IL-6 may contribute to lipid utilization, thus enhancing exercise performance. 

Strenuous exercise may stimulate excessive IL-6 secretion, which is reported to induce muscle damage and may be harmful for athlete health [22,24]. However, in the present study, a KD may relieve the damage induced by excessed IL-6 production. In fact, we have reported in our previous study that KD may reduce muscle damage, as evidenced by lowering damage markers such as creatine kinase (CK) and lactate dehydrogenase (LDH) caused by exhaustive exercise [10]. We have also reported that 24 h after exercise, a KD contributed to fast recovery from fatigue, where muscle damage was also relieved [27]. In this vein, the present study purports that KD may contribute to muscle damage prevention and fatigue recovery by adjusting IL-6 secretion profiles. However, we must acknowledge a limitation, since the volume of soleus muscle is limited, we have had to measure mRNA expression or protein production. Our choice to conduct this analysis type was based on a single acute exhaustive exercise test having more potential to alter gene expression on a transcriptional level. Interestingly, the observation on gastrocnemius IL-6 responses indicated that protein production may happen even in such a short period; further studies may focus on protein production responses induced by acute exercise.

### 3.3. Fatty Acid Mobilation-Related Gene Expression after Exhaustive Exercise under Endogenous Ketosis in Epididymal Adipose Tissue

**Figure 3:**
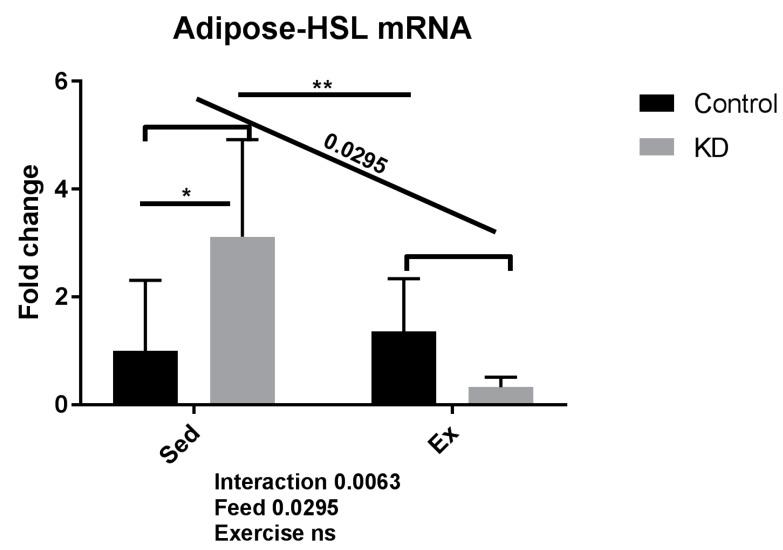
mRNA expression of lipolytic enzyme in adipose tissue immediately after exhaustion. HSL: hormone-sensitive lipase. ns: no significance observed. * *p* < 0.05 and ** *p* < 0.01. Significance between groups are marked using slashes.

As shown in Figure 3, gene expression levels of adipose triglyceride lipase (ATGL) were significantly enhanced by KD in the sedentary subjects, indicating the up-regulated lipid mobilization and utilization is enhanced by KD in sedentary condition in adipocytes. However, KD plus exercise reversed this increase. One plausible explanation for this phenomenon is the lack of plasma IL-6, thus the ability to mobilize fatty acid from adipose tissue is reduced. Adrenergic blocking agents are reported to reduce fatty acid mobilization during fasting, and IL-6 is reported to function as adrenergic hormone [25].

During endurance exercise, the body requires energy. Fatty acids are then released from adipocytes and mobilized for use to provide this energy. When adrenaline or adrenaline-like signaling molecules, such as IL-6, increase and bind to specific receptors on the surface of adipocytes, a cascade of reactions is then induced [25]. Lipases like ATGL start to hydrolyze TG to produce NEFA. Therefore, the increased circulatory NEFAs will be detected as they are transported to skeletal muscle, the heart, and the liver. In the liver, some NEFAs may be resynthesized into triglycerides and then transported by carrier lipoproteins to muscle and other tissues, thus contributing to provide energy to promote exercise capacity [2,3,4]. Accordingly, concentrations of very low-density lipoproteins are much higher at baseline in the KD group, indicating an abundant lipid pool [10,27]. 

ATGL is also known as desnutrin, a patatin-like phospholipase domain-containing protein [30]. Hormone-sensitive lipase (HSL) is also known as cholesteryl ester hydrolase (CEH), another intracellular neutral lipase [31]. ATGL and HSL are both rate-limiting enzymes mediating TG hydrolysis, and play critical roles in mobilizing fatty acids. Briefly, they hydrolyze fatty acids from TG, after which fatty acids of IMTG-origin will be directly used for beta-oxidation, or fatty acids from lipid drop-origin will be transported by lipoproteins such as very low-density lipoprotein (VLDL) from adipose tissue into muscle fibers during exercise [32]. Interestingly, adipocyte-specific ATGL deficient mice presented lowered capacity toward submaximal exercise, whereas skeletal muscle-specific ATGL deficient mice preserved submaximal exercise capacity [33]. These results indicate that circulating fatty acids in the blood that come from adipocytes by lipase-mediated lipolysis, also contribute to submaximal exercise performance. The treadmill running protocol in this investigation is similar to the submaximal exercise protocol used in this previous study, therefore suggesting that fat mobilization during exercise is critical. Results of the present study show that KD mice may experience decreased NEFA mobilizing ability from adipose tissue, despite an enhanced exercise capacity; leading us to suspect that IMTG or energy from other metabolites also play dominant roles in this process. Therefore, we conducted transcriptional analysis of ketolysis, lipolysis and FAO in skeletal muscle to explore these responses.

### 3.4. Ketolytic Gene Expression after Exhaustive Exercise under Conditions of Endogenous Ketosis in Both Slow- and Fast-Twitch Muscles

**Figure 4:**
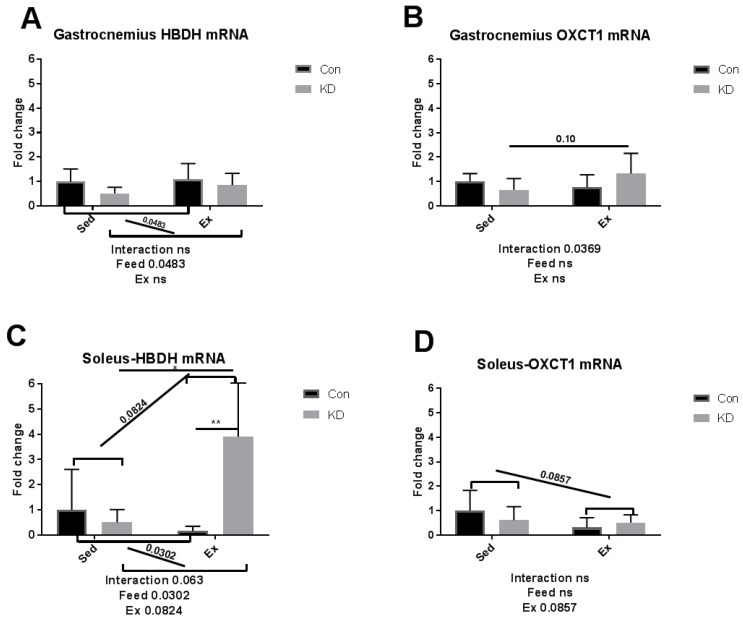
mRNA expression of ketolytic rate-limiting enzyme immediately after exhaustion in gastrocnemius (**A**,**B**) and soleus (**C**,**D**). HBDH: hydroxybutyrate dehydrogenase. OXCT-1: 3-oxoacid CoA-transferase. ns: no significance observed. * *p* < 0.05 and ** *p* < 0.01. Significance between groups are marked using slashes.

**Figure 5:**
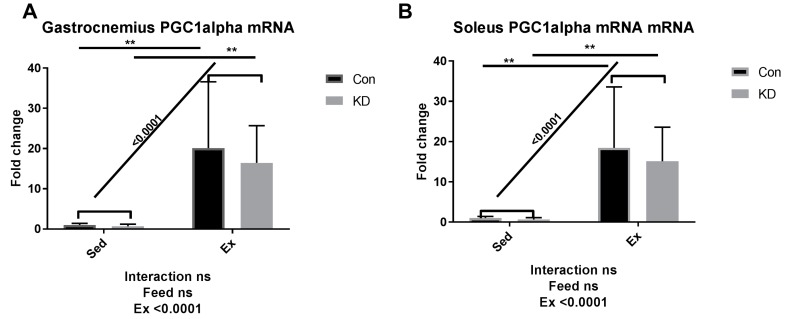
mRNA expression of peroxisome proliferator-activated receptor gamma coactivator-1alpha in gastrocnemius (**A**) and soleus (**B**). ns: no significance observed. ** *p* < 0.01. Significance between groups are marked using slashes.

As we reported previously, an eight-week KD enhanced exercise capacity in KD + Ex subjects, ketone bodies were dramatically consumed during an exhaustive exercise, and plasma β-HB concentrations dropped from 2.4 ± 0.64 mmol/L to 0.72 ± 0.10 mmol/L, indicating enhanced ketolysis. In the present study, we examined whether key enzymes during ketolysis were altered by a KD. As shown in Figure 4A–D, gene expression levels of ketolytic enzymes were altered by KD or exercise in a fiber-specific manner. Figure 4A,C show the change of a rate-limiting enzyme, hydroxybutyrate dehydrogenase (HBDH), in both fiber types. In white fast-twitch muscle fiber (gastrocnemius), HBDH mRNA was down-regulated by KD (Figure 4A), however in red slow-twitch muscle fiber (soleus), which has more relevance with endurance capacity, HBDH mRNA expression was significantly up-regulated (Figure 4C). Figure 4B,D show the change of another rate-limiting enzyme during ketolysis, 3-oxoacid CoA-transferase (OXCT)-1, a rate-limiting enzyme during ketolysis, in both fiber types [34]. Gastrocnemius OXCT-1 mRNA expression was not altered by KD or exercise (Figure 4B), while soleus OXCT-1 mRNA expression was decreased by exercise in the sedentary group (Figure 4D). The above results indicated that soleus HBDH may be the key enzyme that contribute to ketolysis, thus contributing to endurance exercise capacity. Peroxisome proliferator-activated receptor gamma coactivator (PGC)-1alpha expression was dramatically enhanced by exercise, but a KD did not influence this response (Figure 5).

Red slow-twitch muscle like the soleus plays a key role in endurance exercise, whereas a white fast-twitch muscle like the gastrocnemius plays a secondary role [20]. Thus, in the previous and present study, a probable mechanism is that the expression of HBDH, a key enzyme during ketolysis, was enhanced in red, slow-twitch muscle, therefore contributing to promote ketolysis and exercise capacity.

Ketolysis is a complete oxidation process of ketone bodies whilst ketone bodies are utilized by mitochondria of extrahepatic tissues via a series of enzymatic reactions. Ketolysis is regulated by a rate-limiting OXCT-1 and HBDH. Therefore, we measured the transcriptional alternation of these enzymes in different muscle tissues. In our previous study, plasma ketone body increased rapidly in the sedentary KD group. However, after exhaustive exercise, plasma ketone bodies of those KD + Ex mice dropped dramatically compared with those in the Con + Ex group, suggesting that ketone bodies were a primary energy source in the KD + Ex group [10]. These results indicated that an eight-week KD improves ketolysis, the ability for subjects to utilize ketone bodies. Therefore, to investigate the mechanism of this enhancement, we assessed key enzymes involved in ketolysis in both fiber types.

Results here indicated that HBDH plays the key role in the improvement of exercise capacity after an eight-week KD. It is reported that elevation of PGC-1alpha levels in skeletal muscle may improve systemic ketolytic capacity [35]. Since PGC-1alpha mRNA expression was not significantly influenced by KD, the enhanced ketolysis capacity may be merely attributed to the abundance of available ketone bodies, without enhancement of basal metabolic rate mediated by PGC-1alpha.

### 3.5. Lipolysis- and Fatty Acid Oxidation-Related Gene Expression after Exhaustive Exercise during Endogenous Ketosis in Both Slow- and Fast-Twitch Muscles

As we reported previously, an eight-week KD enhanced exercise capacity in KD + Ex subjects, where free fatty acids were dramatically consumed during an exhaustive exercise [10]. Plasma NEFA dropped from 2.2 ± 0.65 µEq/L to 1.5 ± 0.43 µEq/L, indicating an enhanced FAO [10]. In the present study, we observed whether key enzymes during FAO were altered by a KD.

**Figure 6:**
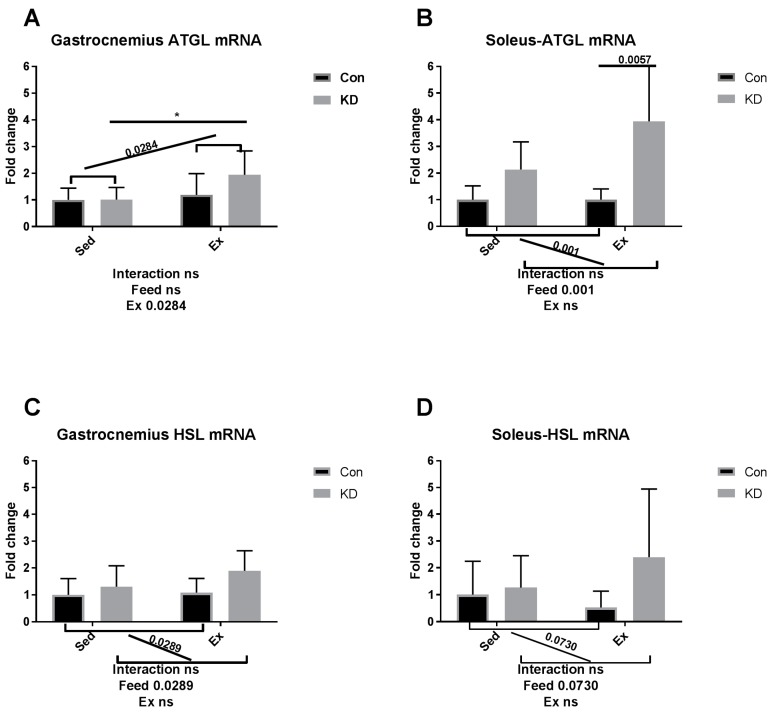

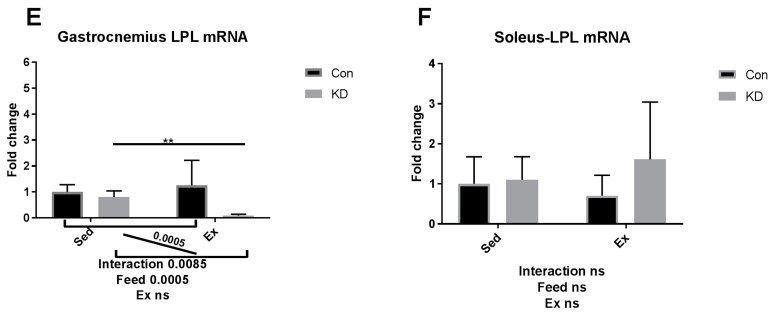
mRNA expression of lipolytic enzymes immediately after exhaustion in gastrocnemius (**A**,**C**,**E**) and soleus (**B**,**D**,**F**). ATGL: adipose triglyceride lipase. HSL: hormone-sensitive lipase. LPL: lipoprotein lipase. * *p* < 0.05 and ** *p* < 0.01. Significance between groups are marked using slashes.

As shown in Figure 6A–D, though gastrocnemius ATGL mRNA was enhanced by exercise between KD and KD + Ex groups (Figure 6A), other results indicated that ATGL mRNA expression in soleus, and HSL mRNA expression levels in both fibers were up-regulated by KD, but not by exhaustive exercise (Figure 6B–D). The above results are observed in both muscle types. Since adipose ATGL mRNA expression appears to be unrelated with KD plus exercise, enhanced mobilization of intramuscular fatty acid may be the main factor relating to an enhanced exercise capacity. As shown in Figure 6E,F, lipoprotein lipase (LPL) mRNA expression pattern in the gastrocnemius was the same as lipase mRNA expression in adipose tissue. To be specific, LPL mRNA expression was significantly decreased in the KD + Ex group, compared to that in the KD group (6E). Soleus LPL mRNA expression was not altered by KD or exercise (6F). Taken together, the reduced fatty mobilization from adipocytes partly from higher blood NEFA and TG, accompanied by an enhanced fatty acid availability from the IMTG pool, may have reduced the need for LPL. 

**Figure 7:**
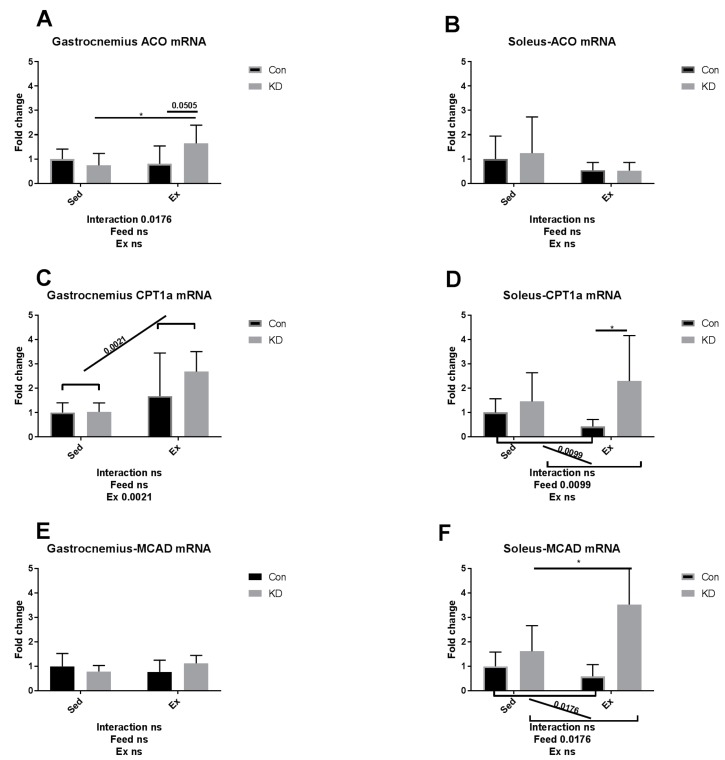

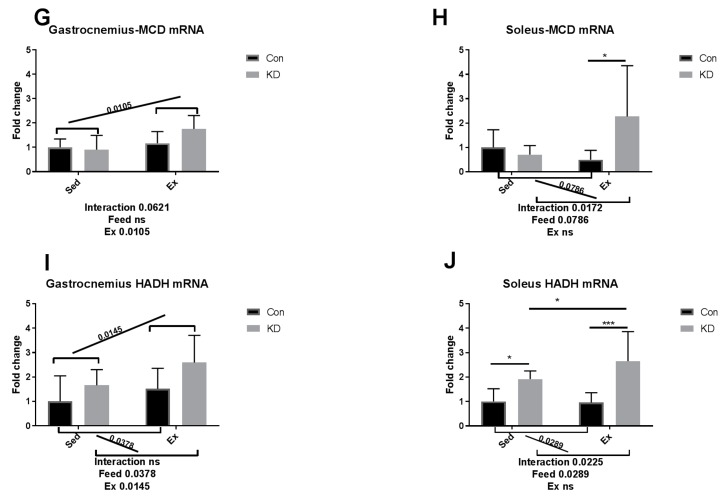
mRNA expression of fatty acid oxidation-related enzymes immediately after exhaustion in gastrocnemius (**A**,**C**,**E**,**G**,**I**) and soleus (**B**,**D**,**F**,**H**,**J**). ACO: acyl-CoA oxidase. CPT-1 alpha: carnitine palmitoyl transferase–1 alpha. MCAD: medium chain acyl-CoA dehydrogenase. MCD: malonyl-CoA decarboxylase. HADH: hydroxyacyl-coenzyme A dehydrogenase. ns: no significance observed. * *p* < 0.05 and *** *p* < 0.001. Significance between groups are marked using slashes.

As shown in Figure 7A,C,I in white, fast-twitch muscle, carnitine palmitoyl transferase (CPT)-1alpha, acyl-CoA oxidase (ACO) and hydroxyacyl-coenzyme A dehydrogenase (HADH) mRNA expression levels were enhanced by a KD after exhaustive exercise. As shown in Figure 7D,F,H,J in red slow-twitch muscle, the diet played as a main factor regulating FAO. CPT-1alpha, medium chain acyl-CoA dehydrogenase (MCAD), malonyl-CoA decarboxylase (MCD) and HADH mRNA expression levels were all enhanced. Exercise also enhanced HADH mRNA expression (Figure 7I,J), indicating that HADH activity may be enhanced by exercise alone. The results combined indicate that KD contributes to an enhanced mRNA expression of FAO-related enzymes.

In an article published several years ago, the authors metaphorically, and appropriately, called adipose ATGL and HSL, “*the movers and shakers of muscle lipolysis*” [36]. After reaching the working site, muscle LPL hydrolyzes very low-density lipoprotein and harvests fatty acids, which will be finally utilized as fuel. CPT-1alpha, ACO, HADH, MCAD and MCD are key regulating enzymes during fatty acid beta-oxidation [37]. For IMTG, the fatty acids are harvested by intramuscular lipase ATGL and HSL and then hydrolyzed by the above enzymes [38]. Therefore, it may be important to increase intramuscular enzymatic activity to enhance exercise capacity. Several nutrients have been reported to possess exercise capacity-enhancing properties, but their mechanisms of action are hardly reported [39,40]. For example, green tea extract supplementation contributed to a higher muscle beta-oxidation activity, lower malonyl-CoA content and higher glycogen content [41,42]. Whilst the macro nutrition profile in our study is different from the above study, the mechanisms of actions are partially similar. In our previous study, we suggested that a combined use of anti-oxidants and a KD should be implemented to ameliorate or diminish the excess oxidation that may be caused during exercise by KD. Green tea extract seems to be a powerful co-supplementation, with proven anti-oxidative and anti-inflammatory properties [10,43,44]. A recent study, using polyphenols as an exercise supplementation, also detected those enzymes noted above [45]. However, though supplementation successfully enhanced exercise capacity, it failed to alter enzymatic response during FAO. Micro-nutrients may be too weak to alter the FAO profile, but the FAO profile was altered here by an eight-week KD.

Recently, a research team from Japan reported that a 12-week KD managed to alter FAO-related mRNA expression, including CPT-1beta and HADH, enhancing FAO capacity in both heart and muscle tissues [46]. Our studies have also confirmed that a KD is able to accelerate FAO, thus contributing to exercise capacity. However, when considering the potential adverse effect of a HFD, shorter periods involving a KD may be preferred. 

### 3.6. Glycerol Profile after Exhaustive Exercise during Endogenous Ketosis

**Figure 8:**
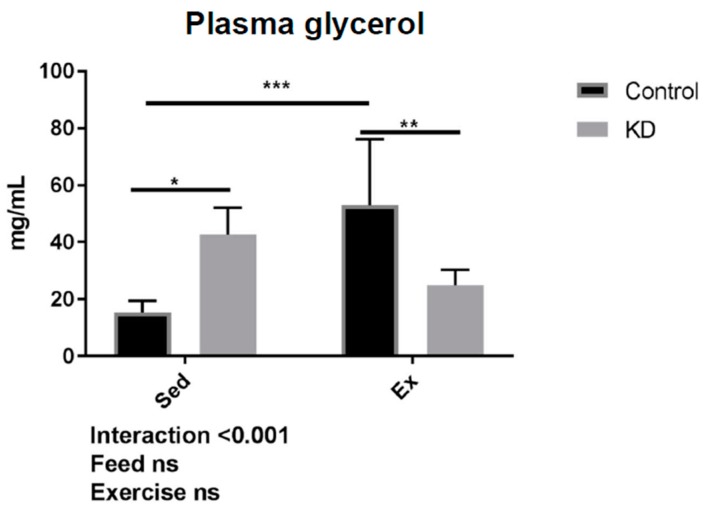
Plasma glycerol concentration after exhaustion. ns: no significance observed. * *p* < 0.05, ** *p* < 0.01, *** *p* < 0.001.

**Figure 9:**
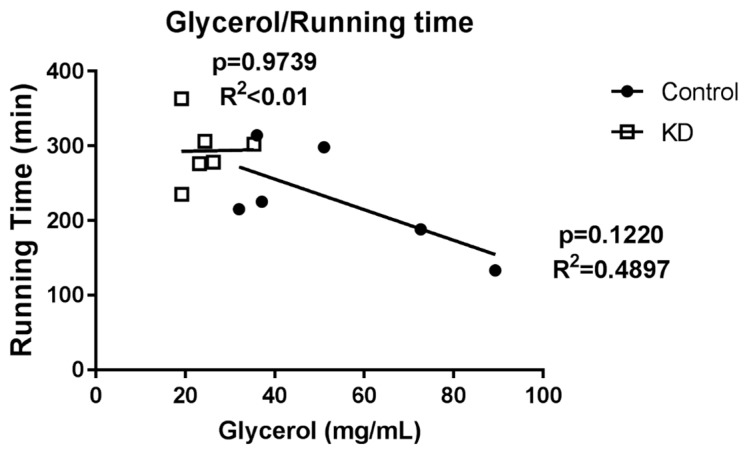
Correlation of plasma glycerol and exercise capacity. No significance was observed.

As shown in Figure 8, in the KD + Ex group, a significant decrease in blood glycerol content was observed after exhaustive exercise. Interestingly, this effect was different in the control group. We also observed a relationship between glycerol concentration and running time. However, as shown in Figure 9, no significance was obtained. 

Glycerol supplementation was found to have an effect on aerobic and anaerobic performance, and other health purposes [47]. TG hydrolyzation is increased during exercise due to an enhanced energy need, whilst this hydrolyzation may also be increased by the need to maintain basal metabolism in a KD group where fat is the primary fuel. This may explain why at baseline, glycerol concentrations are higher, and why glycerol concentrations were increased significantly by exhaustive exercise in the Con + Ex group; a response was also reported by another study [45]. During biological esterification, glycerol is esterified with free fatty acids to form new TG molecules. Glycerol is also used for gluconeogenesis to promote glucose concentrations and maintain basal metabolism. During ketosis or exhaustive exercise, systemic gluconeogenesis and re-lipogenesis are both required, thus contributing to the hypothesis that glycerol will be highly consumed in the KD + Ex group. It may be concluded that gluconeogenesis and re-lipogenesis are more flexible in KD subjects, indicating an enhanced metabolic flexibility resulting from a KD.

### 3.7. A Comprehensive Summary of this Study

In our previous studies, we have reported that a KD can enhance exhaustive exercise capacity and a general metabolic profile during exhaustive exercise [10]. We presumed that this endurance enhancement may be attributed to enhanced ketolysis and lipolysis. Furthermore, we have previously reported that a KD may enhance plasma NEFA concentration restoration over 24 h recovery, after exhaustive exercise [27]. Therefore, when combined with the present results, we postulate that an eight-week KD may enhance “metabolic flexibility”. 

Metabolic flexibility is used as a term to describe the ability to adapt to conditional change in metabolic demand. An eight-week KD helped establish a lipid-focused metabolic system through keto-adaption, thus increasing metabolic flexibility [48]. This concept should not negate the conception of “glycogen loading” before competitions, but an adequate KD may help our body be more flexible during the choice of fuel usage during exercise. Compared to a glucose-centered metabolic system, a long-term KD feeding leads to an establishment of a fatty acid oxidation-centered metabolic system. Moderate training may enhance the ability to utilize ketone bodies as well as fatty acids, or increase fatty acid mobilization from adipose tissue to the circulation. 

We have also reported that an eight-week KD may increase intramuscular oxidative stress [27]. As discussed above, certain supplementation such as green tea extract or polyphenols may alleviate exhaustive exercise-induced oxidative stress, and those anti-oxidants may then contribute to promote muscle health and exercise capacity [43,44,45]. Further investigations examining these associations are required. 

## 4. Conclusions

We report that an eight-week KD enhanced exercise capacity, and suggested that mechanism involved in this response may be enhanced lipolysis and ketolysis; according to the metabolite profile observed before and after exercise. In the present study, we also investigated critical mRNA expression during fatty acid mobilization, FAO and ketolysis. We found that an eight-week KD may remodel the lipid metabolism profile, thus contributing to enhance exercise capacity. Furthermore, we found that a KD may alter the IL-6 synthesis and secretion profile, thus contributing to FAO and muscle damage prevention. Importantly, both the lipid metabolism and IL-6 secretion responses appear to have muscle fiber specificity. Taken together, the previous and present results revealed that an eight-week KD may: (1) enhance exercise performance by up-regulating ketolysis and FAO ability, and (2) have potential to prevent muscle damage by altering the IL-6 secretion profile. Therefore, a KD may be a promising diet approach for endurance sports and injury prevention.
